# Chronic exposure to a common azole fungicide is associated with reactivation of chronic *Plasmodium* infections in farmland birds

**DOI:** 10.1016/j.ijppaw.2026.101222

**Published:** 2026-03-27

**Authors:** Romain Pigeault, Coraline Bichet, Pauline Bellot, François Brischoux, Clémentine Fritsch, Frédéric Angelier

**Affiliations:** a Laboratoire Ecologie et Biologie des Interactions, UMR-CNRS 7267, Université de Poitiers, France; b Centre d’Etudes Biologiques de Chizé, UMR-CNRS 7372, La Rochelle Université, Villiers-en-Bois, France; c Laboratoire Chrono-Environnement, UMR 6249 CNRS, Université Marie et Louis Pasteur, Besançon, France

**Keywords:** Avian malaria, Pesticide, Tebuconazole, Chronic infection, Blood parasites, Haemosporidians

## Abstract

Over the past decades, the widespread use of pesticides has raised concerns about their effects on non-target organisms. Among pesticides, fungicides remain comparatively understudied, despite representing nearly 40% of total pesticide sales in Europe. Wild birds living in or close to farmlands are frequently exposed to tebuconazole, a triazole fungicide widely used in agroecosystems. While its effects on avian physiology and life-history traits have been the subject of several studies, the downstream effects that this agrochemical may exert on host–parasite interactions remain poorly understood. Here, we investigated whether chronic exposure to tebuconazole alters the dynamics of avian malaria chronic infection in house sparrows (*Passer domesticus*). Wild-caught adults were maintained under semi-natural conditions and malaria infection was monitored before and after an eleven-week exposure period to tebuconazole ingestion, starting in late autumn and ending in mid-winter. At the beginning of the experiment, 70% of the birds were chronically infected with malaria. Then, while prevalence declined in control individuals during winter, as typically expected in passerines living in temperate climates, it increased by 20% in tebuconazole-exposed individuals. No significant difference in parasite load was observed between control and exposed birds, suggesting that tebuconazole did enhance parasite replication rate but may instead have promoted the reappearance of parasites from exo-erythrocytic stages. Our findings highlight that exposure to tebuconazole can influence chronic malaria dynamics. Future work should assess how combined exposure to multiple pesticides and environmental stressors might exacerbate these effects, with broader implications for host–parasite interactions in agricultural landscapes.

## Highlights

• Chronic exposure to tebuconazole altered avian malaria infection dynamics in birds.
• Malaria prevalence declined in controls but rose by 20% after fungicide ingestion.
• Parasite load was unchanged, suggesting reactivation of chronic infections.
• Tebuconazole can affect host–parasite interactions in agroecosystems.

## Graphical abstract

**Figure:**
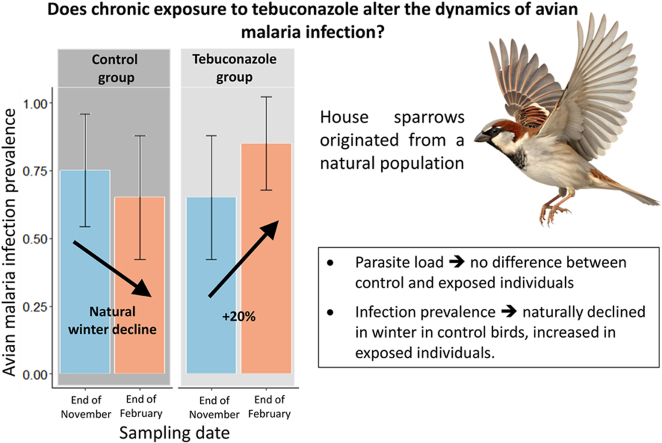

## 1 Introduction

Rapid human population growth over the past decades has intensified agricultural practices worldwide, leading to an increased reliance on chemical inputs such as fertilizers and pesticides (Ecobichon, 2001; Fernandez-Cornejo et al., 2014). Although the use of agrochemicals has provided substantial benefits, with pesticides playing a crucial role in reducing agricultural losses (Fernandez-Cornejo et al., 2014; Popp et al., 2013), the regulation and risk assessment of pesticides has constantly been strengthened since the mid last century and numerous countries enforced strategies to reduce their use and associated risks (Sabarwal et al., 2018; Monnet et al., 2026). The widespread application of these chemical inputs has indeed raised serious concerns for both public health and the environment (Devi et al., 2022; Monnet et al., 2026). Nevertheless, knowledge of the adverse effects of chemical inputs is far from uniform across all types.

Among the various types of pesticides, fungicides have historically received less attention than insecticides (Köhler and Triebskorn, 2013; Zubrod et al., 2019). Yet, in the European Union, fungicides accounted for approximately 39% of total pesticide sales in 2023, and in wine-growing regions, they can constitute over 90% of all pesticide applications (source date: Eurostat). The most widely used fungicides include triazoles (e.g., tebuconazole, prothioconazole), strobilurins (e.g., azoxystrobin), succinate dehydrogenase inhibitors (SDHIs), and inorganic fungicides such as copper- and sulfur-based compounds (Triantafyllidis et al., 2022). Fungicides have historically received far less attention than other pesticides partly because of their presumed lower acute toxicity (Köhler and Triebskorn, 2013; Zubrod et al., 2019). This assumption has been increasingly challenged, as several recent studies have shown that both acute (short exposure to very high concentrations) and chronic (longer exposure to lower concentrations) exposure to fungicides - particularly triazoles - can impair the physiology and life-history traits of vertebrate species, especially birds living in agricultural environments (Bellot et al., 2022a, 2023, 2024, 2025; Jiménez-Peñuela et al., 2025; Lopez-Antia et al., 2021). For example, experimental exposure to environmentally relevant concentrations of tebuconazole (i.e., concentrations that can be found in the environment, such as in soil, water or organisms), one of the fungicides to which farmland birds are most frequently exposed (Angelier et al., 2023; Fernández-Vizcaíno et al., 2022), has been shown to reduce breeding success in red-legged partridges (*Alectoris rufa*), notably through lower hatching rates (Lopez-Antia et al., 2021), and to impair offspring growth and survival in house sparrows (*Passer domesticus*) (Bellot et al., 2025). Although the underlying mechanisms remain unclear, several studies have demonstrated moderate but significant effects of triazole fungicides on key physiological parameters. These include reductions in plasma lipid and protein concentrations (Lopez-Antia et al., 2021), as well as significant decreases in the levels of several hormones, such as thyroid hormones (Bellot et al., 2023), oestradiol (Fernández-Vizcaíno et al., 2020), and the androgens testosterone and androstenedione (Poulsen et al., 2015; Machado et al., 2021).

Such physiological effects, in addition to affecting reproduction, could directly or indirectly influence other life-history traits, such as susceptibility to infection by pathogens or the ability to control latent infections (Fritsch et al., 2024). Indeed, growing evidence from invertebrate systems indicates that fungicide exposure can impair immune function and increase disease susceptibility in non-target organisms. In honey bees (*Apis mellifera*), fungicide exposure has been shown to disrupt immune responses and increase viral infection (Khan and Rolff, 2025), while in bumble bees (*Bombus impatiens*), it has been associated with higher gut parasite loads and delayed recovery (Runnion et al., 2025). To date, however, no study has examined the relationship between vertebrate exposure to fungicides and susceptibility to pathogens other than fungi. Yet, fungicide exposure can impair vertebrate host immunocompetence (Bailly et al., 2026), either directly by suppressing immune responses (reviewed in Cestonaro et al., 2022) or indirectly by altering resource allocation strategies (Corvalan et al., 2025).

In this study, we have explored the relationship between avian malaria infection patterns (prevalence and load) and tebuconazole exposure in house sparrows (*Passer domesticus*), a bird species typically associated with agroecosystems. This passerine species is known to host a wide diversity of haemosporidian parasites (Pigeault et al., 2021), including more than 20 distinct lineages of *Plasmodium* (Ferraguti et al., 2023). Infection by this vector-borne disease may negatively affect birds’ life history traits (Grabow et al., 2024; Lachish et al., 2011; Pigeault et al., 2018) and ultimately influence house sparrow population dynamics (Dadam et al., 2019). More specifically, we investigated the relationship between exposure to tebuconazole and the chronic infection dynamic of *Plasmodium* within blood compartment. Many avian malaria lineages are indeed characterized by long-lasting chronic infection stages that can be punctuated by recurrences (relapse or recrudescence, (Applegate, 1971; Cornelius et al., 2014; Pigeault et al., 2024, 2023; Qiu et al., 2025). Although the underlying mechanisms of such brief, but significant, increase in parasite load in bird blood are not yet fully understood, exposure to stress appears as one of the key drivers of recurrence (Cornelius et al., 2014; Pigeault et al., 2023). If ingestion of tebuconazole induces physiological stress, as suggested by previous studies (Bellot et al., 2022b, 2023), one possible outcome is the reactivation of latent *Plasmodium* infections in house sparrows. However, given the limited empirical evidence on the effects of fungicides on vertebrate host–parasite dynamics, alternative outcomes are also plausible, including no effect or even suppression of parasite detectability.

## 2 Material and methods

### 2.1 Experimental design and biological materials

This study is based on an experimental investigation conducted on a colony of captive adult house sparrows originating from the same wild population located in the vicinity of the lab (Secondigné sur Belle) and housed at the ‘Centre d’Etudes Biologiques de Chizé’ (CEBC, Deux-Sèvres, France, Bellot et al., 2024). The sparrows were fed *ad libitum* with a mixture of commercial seeds, millet on the stalk, sand, and mineral/salt blocks. Details of the experimental design and captive conditions are provided in Bellot et al. (2024). Briefly, birds were captured between the 16 and 29 October 2020 and housed in outdoor aviaries (four aviaries in total). The experiment then took place from 23 November 2020 to 23 February 2021, a period during which the absence of mosquito vectors at this latitude made *Plasmodium* (re)infections highly unlikely (Lalubin et al., 2013; Ponçon et al., 2007). Fifteen days after an initial blood sampling take between the 23 and 24 November 2020 (T0, corresponding to the initial sampling time, see below), an interval sufficient for stress induced by handling to no longer significantly affect *Plasmodium* infection dynamics (see Figure 3 in Pigeault et al., 2023), birds were randomly assigned on 8 December 2020 to one of two experimental groups. Twenty birds (8 females and 12 males) were exposed to tebuconazole at a concentration of 550 μg L^−1^ (Sigma-Aldrich, CAS No. 107534-96-3, purity ≥98.0%), administered in tap water provided *ad libitum* for 11 weeks (from 8 December 2020 to 23 February 2021). The remaining twenty birds (10 females and 10 males) served as controls and received tap water without tebuconazole. The duration of 11 weeks was chosen because the use of triazoles in multiple crops often exceeds several weeks. The exposure was stopped after 11 weeks to ensure that the study was conducted during the non-breeding period (the pre-breeding activity starts in March in house sparrows) and that mosquitoes were still inactive at the end of the experiment (Ponçon et al., 2007). The amount of tebuconazole daily ingested is estimated at about 4.1 μg, which corresponds to an exposure concentration of 0.164 mg kg^−1^ bw d^−1^, a concentration 36 times lower than the chronic no observed effect level (NOEL) for birds (Tebuconazole, Ref: HWG 1608, herts.ac.uk). According to previous studies, this experimental exposure to tebuconazole resulted in an ecologically realistic contamination (i.e., 59.7 pg g^−1^ plasma, Bellot et al., 2022b) compared with the concentrations observed in free-ranging birds, such as blackbirds (i.e., mean = 70.7 pg g^−1^ plasma, median = 36.2 pg g^−1^ plasma, Angelier et al., 2023). Importantly, this dose has been shown to induce multiple physiological alterations despite being lower than the NOEL for birds (Bellot et al., 2022b, 2023, 2025).

### 2.2 Blood sampling and measurements

As mention previously, on 23 and 24 November (T0, corresponding to the initial sampling time), blood samples were collected from each of the 40 birds. Blood samples (100 to 150 μl) were collected by puncturing the brachial vein using 25G needles and heparinized micro-hematocrit capillaries. At the end of the exposure period (23 February 2021, T1, corresponding to the second sampling time), a second blood sample was collected from all birds (n = 40). All blood samples were centrifuged (5 min at 7500 rpm), and plasma and blood cells were separated and stored at −20 °C until analysis. Blood cells were used to assess *Plasmodium* prevalence (i.e., proportion of infected individuals relative to the total number of birds sampled at a specific time point) and infection intensity (*Plasmodium* load). At T0 and T1, all control and exposed birds were weighed using a digital scale (±0.1 g). At T0, the tarsus of all birds was measured using a caliper (±0.01 mm). Then, we calculated the Scaled Mass Index (SMI; Peig and Green, 2009), a body condition index that standardizes an individual's mass to a reference body size, using tarsus length at T0 as the morphological measure.

### 2.3 Molecular diagnosis and quantification of *Plasmodium* in bird blood

Quantitative PCR (qPCR) was used to simultaneously assess infection prevalence (presence/absence) and quantify *Plasmodium* DNA in bird blood (*Plasmodium* load). Compared with traditional methods such as microscopy or conventional nested PCR, qPCR offers greater accuracy and sensitivity, particularly during the chronic stage of infection (Ishtiaq et al., 2017; Wardjomto et al., 2023). DNA was extracted from bird blood (n = 40) using the DNeasy Blood & Tissue Kit (Qiagen) according to the manufacturer's instructions. For each individual, two qPCRs were performed: one targeting the *Plasmodium* nuclear cyt *b* gene (primers: L4050Plasmo, 5′-GCTTTATGTATTGTATTTATAC-3′; H4121Plasmo, 5′-GACTTAAAAGATTTGGATAG-3′) and the other targeting the avian 18S rRNA gene (primers: Plasmo18S-f, 5′-GGCAGCTTTGGTGACTCTAGA-3′; Plasmo18S-r, 5′-AGTTGATAGGGCAGACATTCG-3′). Reactions were run with Luna® Universal qPCR Master Mix, and all samples were analyzed in duplicate. One sample with a Ct difference >0.5 between replicates was reanalyzed. Parasite load was expressed as relative quantification (RQ) values. RQ can be interpreted as the fold-amount of target gene (*Plasmodium* 18S rDNA) with respect to the amount of the reference gene (avian 18S rDNA) and is calculated as 2^-(Ct_*Plasmodium*^ ^– Ct_bird)^. For convenience, RQ values were standardized by × 10^4^ factor. Although this was not the primary objective of the study, all samples were also analyzed using the nested PCR method developed by Hellgren et al. (2004) to detect *Leucocytozoon* and *Haemoproteus*/*Plasmodium* infections. The results of this nested PCR show that no birds were infected with *Leucocytozoon* and confirm that qPCR was significantly more sensitive for detecting *Plasmodium* infections than nested PCR (45% of samples testing positive by nested PCR compared to 72% by qPCR). We therefore decided to focus our study on data acquired using qPCR.

### 2.4 Statistical analyses

All statistical analyses were carried out using R (v. 4.1.3). The raw data and the R script used for the analyses and to produce the figures are available on the figshare website (https://figshare.com/s/165b7ecb100b2551665f).

To investigate the effect of tebuconazole treatment on the detectability of *Plasmodium* in bird blood (i.e., infection prevalence), we used a mixed-effects model with a binomial error structure (lme4 package, Bates et al., 2015) and prevalence data were therefore coded as binary data (0: *Plasmodium* not detected, 1: *Plasmodium* detected). Individual bird identity was included as a random factor, nested within aviary, to account for the non-independence of birds housed in the same aviary, as all birds were sampled twice (T0–T1). Sampling date (either December 8, 2020, hereafter referred to as T0, and February 23 or 24, 2021, referred to as T1) was included as a fixed factor, along with sex, scaled mass index (SMI), and treatment (control or tebuconazole-exposed). The interaction between treatment and sampling date was included to test whether the change in infection prevalence over time differed between groups. The interaction between treatment and sex was also included to test whether the effect of tebuconazole treatment on *Plasmodium* infection vary according to bird sex. When the interactions were significant, we used estimated marginal means obtained with the *emmeans* package (Lenth, 2017) to perform post-hoc contrasts on the fitted model. This approach allows testing the effect of sampling date or sex within each treatment group directly from the full mixed model, while properly accounting for the random structure and all other covariates. A generalized linear model was also performed to test whether the infection prevalence was significantly identical in the two groups of birds at the beginning of the experiment. The analysis was carried out on a subset of the dataset containing only the data collected at the start of the experiment (T0). Sex, treatment (control or exposed), and SMI were used as fixed factors in the model. The same analysis was then performed again, this time selecting only the data collected at the end of the experiment (T1).

To assess the effect of tebuconazole exposure on *Plasmodium* load in the blood (i.e., also known as parasitaemia), the analysis was restricted to individuals that were detected as infected at least once during the experiment (i.e., birds that were uninfected both at T0 and T1 were excluded [number of birds included in the analysis: N = 15 for control and N = 17 for treated birds]). A mixed-effects model with a normal error structure was used, with individual birds fitted as a random factor and nested within aviaries. Treatment (control or exposed), sex, sampling date and SMI were included as fixed factors. The interaction between treatment and sex and between sampling date and treatment were also included as fixed factors. Parasite load data were transformed using a Box–Cox procedure to improve normality, with the optimal lambda parameter chosen to best satisfy the assumption of normally distributed residuals. A linear model was also performed to test whether the intensity of infection was identical in the two groups of birds at the beginning of the experiment. The analysis was carried out on a subset of the dataset containing only the data collected at the start of the experiment (T0). The sex, the group to which the birds belonged (i.e., control and those later exposed to tebuconazole), and their SMI were used as fixed variables in the model.

Maximal models, including all two-way interactions, were initially fitted to the data. Higher-order (three-way) interactions were not tested in order to retain sufficient statistical power, given the limited sample sizes. The significance of explanatory variables was assessed using the drop1 function (package lme4, Bates et al., 2015) with a likelihood ratio test (approximated by a chi-square distribution, Bolker, 2008) for mixed models and GLM and a F-test for LM. Significant test values reported in the text correspond to the minimal model, whereas nonsignificant results refer to tests conducted prior to term deletion. Model assumptions were verified using the simulateResiduals and testDispersion function from the DHARMa package (Hartig and Lohse, 2024). This approach allowed us to assess residual uniformity, overdispersion, zero inflation, outliers, and overall model fit for binomial and Gaussian models. This diagnostic procedure was applied to all models described below and no violations of model assumptions were detected.

## 3 Results

**Fig. 1:**
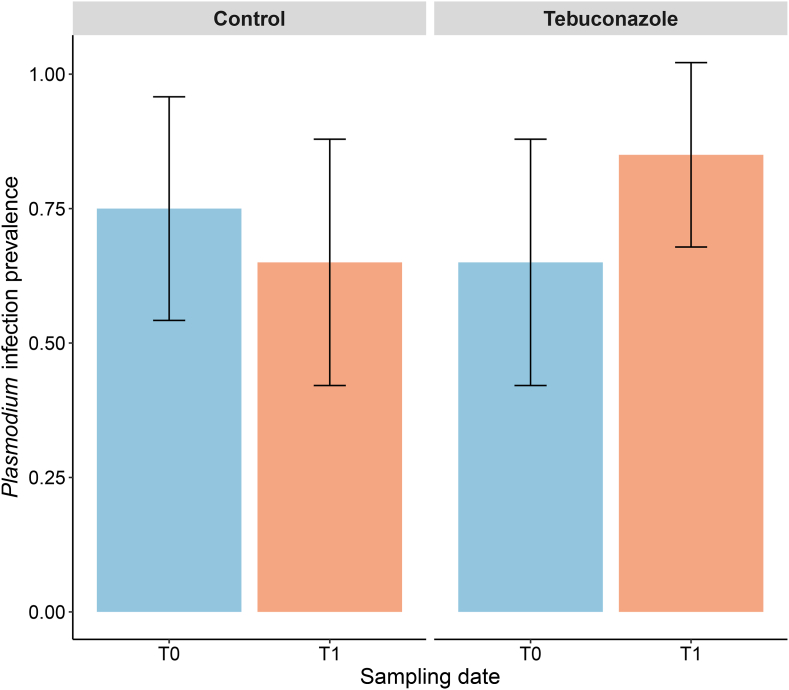
Change in infection prevalence over the course of the experiment according to birds' exposure status to tebuconazole. Error bars represent the 95% confidence interval. Control: unexposed to Tebuconazole, Tebuconazole = exposed to Tebuconazole. Blue (T0) corresponds to the first measurement at the start of the experiment (8 December 2020), and orange (T1) corresponds to the second blood sampling conducted 11 weeks later (23 February 2021).

At the start of the experiment (i.e., 23 and 24 November 2020), *Plasmodium* infection was detectable by qPCR in 70% of the birds (95% CI: 55%–84%). The proportion of infected individuals was similar between birds in the control group and those that would later be exposed to tebuconazole (LRT = 1.105, p = 0.293). At the end of the experiment (i.e., 23 February 2021) the percentage of birds detected as infected was 75% (95% CI: 61%–89%) and was not different between the two groups of birds (LRT = 2.473, p = 0.116). However, when examining changes in infection prevalence between the beginning and the end of the experiment, a significant interaction was observed between sampling time (T0 or T1) and treatment (Fig. 1; LRT = 25.29, p < 0.0001). In control birds, infection prevalence decreased by 10% during the experiment (December: 0.75, 95% CI: 0.54–0.95; February: 0.65, 95% CI: 0.42–0.87, z ratio = 3.57, p = 0.0008), whereas in tebuconazole-exposed birds, prevalence increased by 20% (December: 0.65, 95% CI: 0.42–0.87; February: 0.85, 95% CI: 0.68–1.0, z ratio = −3.26, p = 0.0012). The interaction between sex and treatment did not have any effect on *Plasmodium* prevalence (LRT = 0.01, p = 0.976). No effect of sex alone or SMI on infection prevalence was observed (LRT = 0.01, p = 0.929; LRT = 0.00, p = 0.986, respectively).

**Fig. 2:**
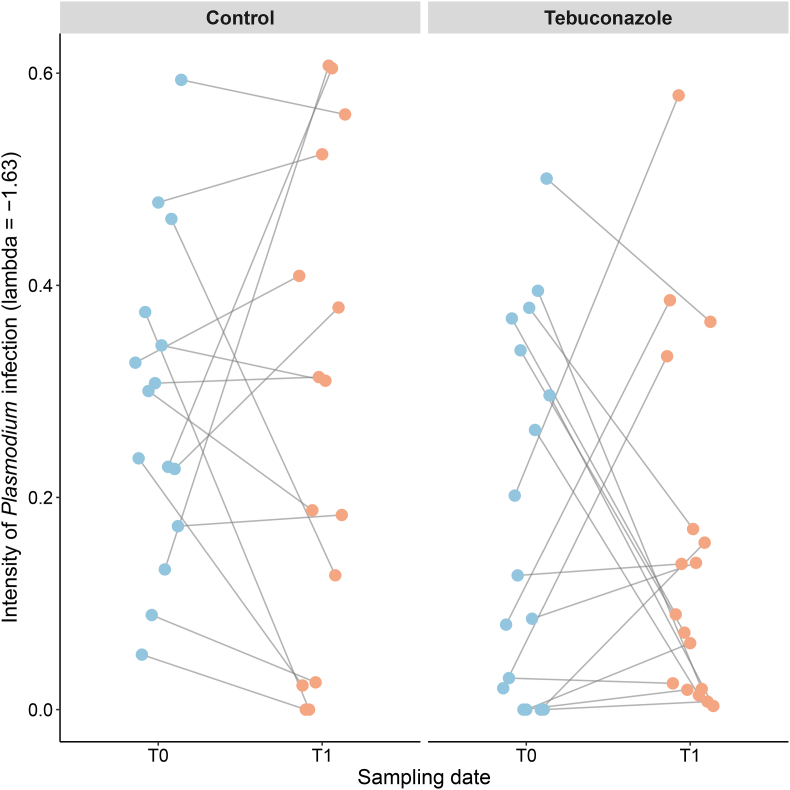
No significant variation in *Plasmodium* load over the course of the experiment. Control: unexposed to Tebuconazole, Tebuconazole = exposed to Tebuconazole. Blue (T0) corresponds to the first measurement at the start of the experiment (8 December 2020), and orange (T1) corresponds to the second blood sampling conducted 11 weeks later (23 February 2021). Each pair of points connected by a line represents repeated measures taken on the same bird. Values of parasite load (RQ) were transformed using a Box–Cox transformation (λ = −1.63; see Statistical analysis section).

As expected during the chronic phase of *Plasmodium* infection at the beginning of the winter period, parasite load in the blood of infected individuals at T0 was extremely low (December: RQ = 0.68 ± 0.24), and not statistically different between birds in the control group and those that would later be exposed to tebuconazole (LRT = 2.740, p = 0.108). Parasite load did not increase significantly over time (February: RQ = 1.67 ± 0.78; LRT = 0.13, p = 0.713; Fig. 2). Exposure to tebuconazole over the 11-week period did not significantly affect *Plasmodium* load (interaction between time of measurement and treatment, LRT = 0.09, p = 0.760). No effects of sex or SMI were observed (LRT = 0.06, p = 0.807; LRT = 0.565, p = 0.452, respectively).

## 4 Discussion

In this study, we showed that exposing wild-caught birds, maintained under semi-natural outdoor conditions, to environmental concentrations of tebuconazole can alter the dynamics of *Plasmodium* infection. The proportion of birds with detectable *Plasmodium* DNA in their blood increased by 20% following 11 weeks of chronic exposure to tebuconazole, whereas infection prevalence declined by 10% in the control group. Although the number of individuals changing infection status over the course of the experiment was limited, the statistical evidence for this pattern was strong. Indeed, the inference is based on a highly significant interaction between sampling time and treatment, indicating opposite temporal trajectories of infection prevalence in control and exposed individuals.

At the start of the experiment, the prevalence of infection in our semi-natural house sparrow colony was comparable to that reported in nearby wild populations (Bichet et al., 2014; Bonneaud et al., 2006). This outcome is expected, as adults were captured only a few weeks before the start of the experiment and had not been treated with antimalarials. Subsequently, the variations in prevalence observed during the study reflect intrinsic fluctuations in parasite dynamics within the hosts. Although birds were housed outdoors without protection from mosquito bites throughout the experiment, they could not acquire new infections, since at this latitude, mosquito vectors are inactive during winter (Lalubin et al., 2013; Ponçon et al., 2007).

The slight decrease in prevalence observed in control birds over the winter period was expected. In many passerine species, including house sparrows, while prevalence and parasite load peak during the breeding season, a decline is consistently observed during winter (Applegate, 1971; Cosgrove et al., 2008; Neto et al., 2020; Pigeault et al., 2024; Schrader et al., 2003). This decline reflects both the cessation of vector activity, and therefore no new infection, and the fact that malaria parasites in chronically infected hosts either disappear from peripheral blood or reach levels so low that they become undetectable by molecular methods (Applegate, 1971; Cosgrove et al., 2008; Pigeault et al., 2024; Schrader et al., 2003). Nonetheless, *Plasmodium* is rarely cleared from its avian hosts. Instead, parasites can persist as exo-erythrocytic stages in various tissues (Huff, 1957; Valkiūnas and Iezhova, 2017). However, after a period of latency, parasites may reappear in the blood, either through increased replication of low-level chronic infections (recrudescence) or reactivation of dormant tissue stages (relapse). These recurrences are frequently observed during spring in temperate regions, though they can also occur sporadically at irregular intervals (Applegate, 1971; Cosgrove et al., 2008; Neto et al., 2020; Pigeault et al., 2023, 2024; Schrader et al., 2003). Indeed, studies have shown that the induction of stress, whether natural or experimentally induced, can trigger a recurrence (Cornelius et al., 2014; Pigeault et al., 2023). Although the first blood sample may cause stress, the birds were not exposed to tebuconazole until 15 days later, a period known to allow the intensity of the infection to return to its baseline level (Pigeault et al., 2023).

In this study, we show that chronic exposure to tebuconazole over 11 weeks significantly increased the proportion of birds in which a chronic *Plasmodium* infection was detectable. This result corroborates a recent study showing that blood parasite prevalence was higher in wild passerines from conventional than from organic farming systems (Bailly et al., 2026). Several studies suggest that exposure to tebuconazole may affect host immunity, metabolism, and physiology (Bellot et al., 2022b, 2023), such alterations may reduce the ability of birds to control *Plasmodium* infections. Alternatively, the host's physiological response to the agrochemical may indirectly promote the reactivation of dormant parasite stages and/or stimulate their replication rate. The original study, from which the blood samples used for *Plasmodium* detection were collected, reported that ingestion of this fungicide did not influence some parameters of the innate immunity in birds (Bellot et al., 2024). Thus, modifications/alterations of the innate immune system seem unlikely to explain the observed increase in infection prevalence among fungicide-exposed birds compared to controls. However, other documented effects of exposure to triazoles, and particularly tebuconazole, such as alterations in metabolism (Bellot et al., 2022b) or plasma biochemistry parameters (Fernández-Vizcaíno et al., 2020) may compromise host immunocompetence or directly enhance parasite ability to (re-)colonize blood compartment. It should be noted that the absence of a significant difference in parasite load (*i.e.,* proportion of infected red blood cells) between exposed and control groups suggests that parasite replication itself was not directly affected by tebuconazole exposure. The observed effects may also have been influenced by the seasonal context of the experiment: even under controlled conditions with *ad libitum* food, winter is physiologically demanding for temperate passerines, and the combined energetic costs of thermoregulation and contaminant detoxification could have transiently constrained resources allocation, subtly affecting host–parasite interactions. Overall, our result suggests that exposure to this fungicide may influence the ability of *Plasmodium* exo-erythrocytic stages to recolonize the blood compartment.

One of the strengths of our study, working with wild-caught birds, also represents a limitation, as we cannot determine the extent to which individuals had been previously exposed to fungicides or other agrochemicals. Although all birds originated from the same population and were randomly assigned to experimental groups, we cannot exclude potential carry-over or synergistic effects between prior exposure to multiple compounds and the experimental exposure to tebuconazole during the study. Another limitation of our study is that it focuses exclusively on *Plasmodium*. Other parasites could also be influenced by fungicide exposure, and coinfections within the blood compartment or elsewhere in the host may affect overall health and immune function, potentially influencing the observed patterns. Moreover, different *Plasmodium* lineages may exhibit distinct dynamics and host interactions, which were not considered here. Our study therefore highlights the need for future research examining the effects of fungicides on entire parasite communities.

To conclude, although the direct effects of chronic exposure to tebuconazole on *Plasmodium* infection observed in this study may appear limited (prevalence increased in exposed sparrows but there was no obvious effect of exposure to tebuconazole on parasite load), they raise broader concerns about the potential indirect ecological consequences of pesticide exposure. It would now be important to extend this type of study to other bird species with different ecological and nutritional niches. Indeed, species identity and diet probably influence their exposure to fungicides and the accumulation of these chemicals. House sparrows are mainly granivorous, while omnivorous species such as blackbirds and starlings consume earthworms and other invertebrates, which can bioaccumulate pesticides (Harris et al., 2000; Tison et al., 2024). Therefore, dietary differences may modulate internal fungicide concentrations and their effects on host-parasite interactions. Moreover, in natural environments, wild birds are rarely exposed to a single compound in isolation but instead face complex mixtures of pesticides and herbicides, combined with other environmental stressors such as food limitation, habitat degradation, and climate irregularities. Testing how such combined pressures affect the dynamics of *Plasmodium* infections would provide valuable insights into the ecological and evolutionary consequences of anthropogenic change for host–parasite interactions, with possible implications for both biodiversity conservation and ecosystem health.

## CRediT authorship contribution statement

**Romain Pigeault:** Writing – review & editing, Writing – original draft, Formal analysis, Data curation. **Coraline Bichet:** Writing – review & editing, Writing – original draft, Methodology, Investigation, Formal analysis, Data curation, Conceptualization. **Pauline Bellot:** Writing – review & editing, Methodology, Investigation, Conceptualization. **François Brischoux:** Writing – review & editing, Methodology, Investigation, Conceptualization. **Clémentine Fritsch:** Writing – review & editing, Methodology, Investigation, Conceptualization. **Frédéric Angelier:** Writing – review & editing, Writing – original draft, Validation, Supervision, Methodology, Investigation, Funding acquisition, Data curation, Conceptualization.

## Data availability statement

Data and scripts are available online: https://figshare.com/s/165b7ecb100b2551665f.

## Conflict of interest

We declare we have no competing interests.
